# Presentation of Trauma Disorder in Adults with Intellectual Disability

**DOI:** 10.1192/j.eurpsy.2023.97

**Published:** 2023-07-19

**Authors:** J. McCarthy

**Affiliations:** King’s College London, London, United Kingdom

## Abstract

**Abstract: Background:**

In the 20 years since a review by McCarthy (2001) on the presentation of Post-traumatic stress disorder (PTSD) in people with intellectual disability (ID), the understanding of trauma disorder has increased.

**Method & Findings:**

Diagnostic criteria for ID will be presented to set the context. A brief summary of the key literature over the past twenty years will be discussed focusing on the presentation of PTSD in people with ID including the use of ICD-11 criteria. Evaluated tools available to assess the impact of life events and trauma for use in people with ID will be outlined including treatment options. The presentation of trauma disorder will be illustrated through a case study.

**Conclusion:**

Psychiatrists need to be aware that a history of trauma may be an underlying factor in the presentation of people with ID to psychiatric services.

**Reference:**

McCarthy J (2001). Post-traumatic Stress Disorder in People with Learning Disability. Advances in Psychiatric Treatment; 7, pp. 163-169.

**Disclosure of Interest:**

None Declared

